# X-ray Structure and Molecular Dynamics Simulations of Endoglucanase 3 from *Trichoderma harzianum:* Structural Organization and Substrate Recognition by Endoglucanases That Lack Cellulose Binding Module

**DOI:** 10.1371/journal.pone.0059069

**Published:** 2013-03-14

**Authors:** Érica T. Prates, Ivana Stankovic, Rodrigo L. Silveira, Marcelo V. Liberato, Flávio Henrique-Silva, Nei Pereira, Igor Polikarpov, Munir S. Skaf

**Affiliations:** 1 Institute of Chemistry, State University of Campinas–UNICAMP. Cx.P. 6154, Campinas, São Paulo, Brazil; 2 Institute of Physics of São Carlos, University of São Paulo, São Carlos, São Paulo, Brazil; 3 Departamento de Genética e Evolução, Universidade Federal de São Carlos, São Carlos, São Paulo, Brazil; 4 Centro de Tecnologia, Escola de Química, Laboratório de Desenvolvimento de Bioprocessos (LaDeBio), Universidade Federal do Rio de Janeiro, Rio de Janeiro, Rio de Janeiro, Brazil; Bioinformatics Institute, Singapore

## Abstract

Plant biomass holds a promise for the production of second-generation ethanol via enzymatic hydrolysis, but its utilization as a biofuel resource is currently limited to a large extent by the cost and low efficiency of the cellulolytic enzymes. Considerable efforts have been dedicated to elucidate the mechanisms of the enzymatic process. It is well known that most cellulases possess a catalytic core domain and a carbohydrate binding module (CBM), without which the enzymatic activity can be drastically reduced. However, Cel12A members of the glycosyl hydrolases family 12 (GHF12) do not bear a CBM and yet are able to hydrolyze amorphous cellulose quite efficiently. Here, we use X-ray crystallography and molecular dynamics simulations to unravel the molecular basis underlying the catalytic capability of endoglucanase 3 from *Trichoderma harzianum* (ThEG3), a member of the GHF12 enzymes that lacks a CBM. A comparative analysis with the *Cellulomonas fimi* CBM identifies important residues mediating interactions of EG3s with amorphous regions of the cellulose. For instance, three aromatic residues constitute a harboring wall of hydrophobic contacts with the substrate in both ThEG3 and CfCBM structures. Moreover, residues at the entrance of the active site cleft of ThEG3 are identified, which might hydrogen bond to the substrate. We advocate that the ThEG3 residues Asn152 and Glu201 interact with the substrate similarly to the corresponding CfCBM residues Asn81 and Arg75. Altogether, these results show that CBM motifs are incorporated within the ThEG3 catalytic domain and suggest that the enzymatic efficiency is associated with the length and position of the substrate chain, being higher when the substrate interact with the aromatic residues at the entrance of the cleft and the catalytic triad. Our results provide guidelines for rational protein engineering aiming to improve interactions of GHF12 enzymes with cellulosic substrates.

## Introduction

Bioethanol is an attractive renewable fuel that has been produced in large quantities by the alcoholic fermentation of concentrated syrups obtained from sugar cane, corn and other feedstocks in countries such as Brazil and the United States, aiming to supplement and, eventually, to replace fossil liquid fuels. Over the last several years, increasing research efforts have been devoted to the production of the second-generation cellulosic ethanol, in which cellulosic biomass is chemically and enzymatically degraded into soluble fermentable sugars. It is estimated that by introducing cellulosic ethanol technology, the overall production of ethanol could be enhanced by as much as 40% without increasing the crop area [1].

Cellulose is the main component of plant cell wall available for bioconversion. Due to the presence of hemicellulose and lignin in the biomass and the stability of glycosidic bonds in the cellulose and hemicellulose, the degradation of this material in nature is accomplished mostly by the action of enzymes [2]–[4]. However, under industrial settings, the enzymatic catalysis is one of the most expensive steps of the biomass-to-cellulosic ethanol bioconversion process due to the low efficiency and the high cost of currently available enzyme preparates. In order to develop strategies for reducing the costs of this process, extensive efforts have been directed to the study of cellulolytic microorganisms and to investigate the mechanisms of biomass enzymatic hydrolysis, as well as general structural and dynamic properties of glycosyl hydrolases (GHs).

Cellulases from *Trichoderma reesei* fungus are among the most widely studied enzymes for cellulose saccharification, both structurally and functionally. *T. reesei* expresses a variety of cellobiohydrolases and endoglucanases that are, with very few exceptions, composed by two domains, the large catalytic core domain (CCD) and the small cellulose-binding module (CBM), which are connected by a heavily glycosylated polypeptide fragment. It is generally accepted that the CBM recognizes and binds to the cellulose surface, whereas the catalytic process is preceded by the detachment of single polysaccharide chain from the crystalline cellulose and subsequent insertion of the chain into the active site of the enzyme's CCD by means of a still poorly understood mechanism. It is proposed that the cellulases move laterally on the cellulose surface by the interaction of the CBM with the cellulose chains [5]–[8]. Also, it has been proposed that the CBM plays an important role in increasing the affinity of the enzyme by the cellulose and in disrupting crystalline domains of the cellulose, and the lack of CBM in engineered enzymes can even prevent the enzymatic process [9]. Thus, it is important to comprehend function of cellulases that do not contain the CBM domain and still are able to catalyze the cellulose hydrolysis. The endoglucanase 3 (known also as Cel12A [10]) of *Trichoderma reesei* (TrEG3) and *Trichoderma harzianum* (ThEG3) constitute an example of these CBM-less enzymes. As such, these enzymes are considered to be poorly adsorbed on the crystalline surface of the substrate. On the other hand, these enzymes are particularly active in the amorphous regions of cellulose or in the hydrolysis of soluble oligosaccharides [11], which suggests that the catalytic core of these enzymes not only possesses the catalytic functions, but might also bear CBM-like motifs specific for interactions with amorphous regions of the substrate. Nevertheless, there are very little molecular level insights into how the enzymes of Cel12A family are able to recognize, bind, and hydrolyze cellulose chains in the absence of a CBM.

In this study, we report the three-dimensional structure of ThEG3 obtained from X-ray crystallography and investigate enzyme-substrate interactions using molecular dynamics (MD) simulations. In order to elucidate how substrate recognition and binding occur in the absence of a CBM, we determine the main interactions of ThEG3 with oligosaccharides (cellotetraose and cellopentaose) and compare the structure of this enzyme with the CBMs of endoglucanase C from *Cellulomonas fimi* (CfCBM) and the homologous, functionally well-characterized, TrEG3 enzyme [12]–[14]. We identify amino acids at the body of Cel12A that play key roles in substrate binding and, thus, provide a structural basis for the fact that Cel12A enzymes relinquish a cellulose-binding module. Such information could be used for optimizing the enzyme efficiency through protein engineering techniques.

## Materials and Methods

### Experimental

Cloning, expression, purification, and crystallization of ThEG3 were conducted as described [15]. A single ThEG3 crystal was mounted in a cryo-loop containing a crystallization solution mixed with 20% of ethylene glycol. The entire data set was collected at the MX-2 beamline at the Brazilian National Synchrotron Light Laboratory (LNLS), in Campinas, Brazil [16]. The diffraction data was recorded using an MARCCD detector and the data set was processed using the program HKL2000 [17]. The structure of ThEG3 was determined by the molecular-replacement method with the program PHASER [18], using as a search model the structure of Cel12A from *Trichoderma reesei* (TrEG3, PDB id: 1H8V), which shows 83% sequence identity with the target. Structure refinement was performed using PHENIX [19]. Manual rebuilding using COOT [20] and addition of water molecules allowed construction of the final model consisting of two polypeptide chains with 226 residues each in the asymmetric unit cell. The refinement converged at R_factor_ = 18.2% and R_free_ = 22.4%. The data set and refinement statistics are given in Table 1. The final crystallographic model and structure factors were deposited in the Protein Data Bank under PDB code 4H7M.

**Table 1:** Collection of data and refinement statistics.

Diffraction data	
Crystal	EG3
Source	Synchrotron
Wavelength (Å)	1.46
Spatial group	P2_1_2_1_2_1_
Network parameters (Å)	a = 47.54, b = 55.57 e c = 157.26
Resolution (Å)	50–2.07 (2.14–2.07)
Completeness (%)	98.97 (95.74)
I/σI	11.96 (7.72)
Redundancy	6.6 (6.3)
R-sym	0.11 (0.20)

Refinement statistics	
Resolution (Å)	2.07
Number of Unique Reflections	25980
Rwork (%)	18.25
Rfree (%)	22.38
Number of Atoms	3803
rotein	3489
olvent	314
B factor	20.80
rotein	19.90
olvent	30.99
Rmsd bond (Å)	0.007
Rmsd angle (°)	1.04

The values in parentheses are related to the layer of higher resolution

### ThEG3-Substrate Complex Models and Molecular Dynamics

We carried out MD simulations of apo ThEG3 (PDB id: 4H7M, this work) and TrEG3 (PDB id: 1H8V [12]) structures, as well as ThEG3 bound to different oligosaccharides, which have been modeled into the binding cleft of the enzyme on the basis of ThEG3 superposition with available structures of protein-substrate complexes. Control simulations were also performed for the parent structures, CfCBM-cellopentaose crystallographic complex (PDB id: 1GU3 [14]) and Cel12A from *Thermotoga maritma* complexed to a cellotetraose ligand (PDB id: 3AMM [21]). The procedures employed to prepare the systems for the simulations are described below.

At the N-terminal of the TrEG3 structure, there is a cyclic pyroglutamate (PCA), originated from cyclization and condensation of a glutamine residue. This reaction often occurs in fungal extracellular enzymes and is believed to increase enzyme stability toward proteases [12]. Furthermore, TrEG3 is glycosylated at the position of Asn164, with a *N*-acetil-D-glucosamine (NAG) bonded covalently to the Asn164 side chain, interacting with Asn91 of another molecule in the crystallographic asymmetric unit. Given that there is no evidence of TrEG3 dimer formation [12] and that TrEG3 exhibits catalytic activity in the absence of PCA [22], the N-terminal glutamine residue was reconstructed and the NAG molecule removed. We also removed the first six residues of ThEG3 structure (EAEAEF), which were artificially added to the sequence as a linker between the recombinant enzyme and a histidine tail during protein purification [15].

In order to study the mechanisms of substrate recognition by Cel12A, we have constructed three different systems comprised of a small oligosaccharide bound to the ThEG3 catalytic cleft. After alignment with ThEG3 using Multiseq in VMD [23], the coordinates of cellotetraose and cellopentaose ligands were taken from the crystallographic structures of Cel12A from *Thermotoga maritima* (TmEG3) [21] and CfCBM [14], respectively, to position these ligands within the catalytic cleft of ThEG3. In addition to these two complexes, a third model was created by prolonging cellotetraose in the ThEG3 structure by one glycosidic unit, forming the ligand which we called cellopentaose*. The ThEG3-substrate constructs containing the cellotetraose, cellopentaose, and cellopentaose* ligands will hereafter be denoted ThEG3-tt, ThEG3-pt, and ThEG3-pt*, respectively.

One of our goals is to comprehend of the Cel12A-substrate interactions that allow cellulose recognition in the absence of a CBM. The adopted strategy was to compare MD simulations of both ThEG3 and a CBM complexed to cellopentaose in order to identify residues that assume similar roles in the interactions with the substrate in both structures. For that purpose, we have chosen to simulate the CBM of the *C. Fimi* endoglucanase C (CfCBM, PDB id: 1GU3) [14]. As a typical CBM of the family 4 of endoglucanases, CfCBM binds mostly to oligosaccharides and amorphous cellulose (http://www.cazy.org/CBM4.html) [24], similarly to TrEG3, which interacts poorly with the crystalline substrate [11]. The first seven residues of the N-terminal of CfCBM are absent in the crystallographic structure presumably because of their high mobility. In order to model these residues, the coordinates of the backbone of the first five residues of a superimposable structure (CBM of laminarinase from *Thermotoga maritima*, PDB id: 1GUI) were used. The other two missing residues were built using Molden [25].

All the systems underwent the same simulation protocol, described as follows. The structures were then placed in a cubic box of about 80 Å in each direction and hydrated by 15,000 water molecules using Packmol [26], [27], so that the hydration layer around the surface of protein was at least 18 Å thick. For all simulated systems, we added at least 53 chlorine ions and 50 sodium ions, maintaining the systems electrically neutral at a salt concentration of approximately 0.16 M.

The ionization states of ionizable residues (Lys, Arg, His, Asp, and Glu) were determined according to their *pKa* values at neutral pH and the molecular environment (high dielectric constant at the protein surface and low dielectric constant in its interior) using the H++ server [28], [29]. Special attention was paid to the choice of the ionization states of the residues comprising the catalytic triad. Considering the function in the catalytic reaction and the interactions with the substrate, the acid catalyst (Glu201 in ThEG3 and Glu200 in TrEG3) and the auxiliary residue (Asp100 in ThEG3 and Asp99 in TrEG3) were considered protonated, whereas the nucleophile (Glu117 in ThEG3Th and Glu116 in TrEG3) was kept in its charged form. Similarly to the TrEG3 structure [12], the ThEG3 crystallographic structure suggests there are hydrogen bonds between side chains of Glu117 and Asp100 and between Glu201 and its neighboring residue, Glu96. The chosen protonation states are consistent with these interactions and favor the hydrolysis reaction [30].

The energy of the system was initially minimized by 500 steps of the conjugate gradient (CG) method [31], [32] as implemented in NAMD [33] to eliminate bad contacts. After minimization, we performed pre-equilibration runs, following the protocol described elsewhere [34]. From the pre-equilibrated systems, we carried out three independent simulations for the apo ThEG3 and TrEG3 of at least 40 ns, reaching a total of 130 ns simulation time for each apo structure, as well as three independent 40 ns runs for each of the substrate-protein complexes: ThEG3-tt, ThEG3-pt, ThEG3-pt*, TmEG3-tt, and CfCBM-pt, amounting 120 ns simulation time for each liganded system. These simulations are not sufficiently long to sample large amplitude motions of the proteins, but they do capture local fluctuations of the structure and are capable of assessing protein-substrate interaction adequately.

All simulations were performed in the *NPT* ensemble with the NAMD program [33], using periodic boundary conditions. The CHARMM force field was used for the proteins and oligosaccharides [35]–[38] and the TIP3P model was used for water molecules [39]. The temperature and pressure were kept constant at 298 K and 1 bar by means of Langevin dynamics and Nosé-Hoover piston methods [40], [41]. The RESPA multiple-time step algorithm [42] was used with the shortest time step of 2 fs. All bonds involving a hydrogen atom were kept at fixed bond length using SHAKE [43]. A 12 Å cutoff with smooth switching function starting at 10 Å was used for the van der Waals forces, whereas electrostatic forces were treated via the particle mesh Ewald method [44].

The overall stability and the structural relaxation of the enzymes were monitored by computing the time evolution of the root mean square deviation (RMSD) of the protein Cα atoms along the simulations (see Supporting Information). We observed that after few nanoseconds the RMSD of the backbone became stable. For all systems, the first 6 ns of simulation were not considered for calculating average properties. The RMSD values converge to values between 1 and 2 Å.

## Results and Discussion

### ThEG3 3D structure

**Figure 1:**
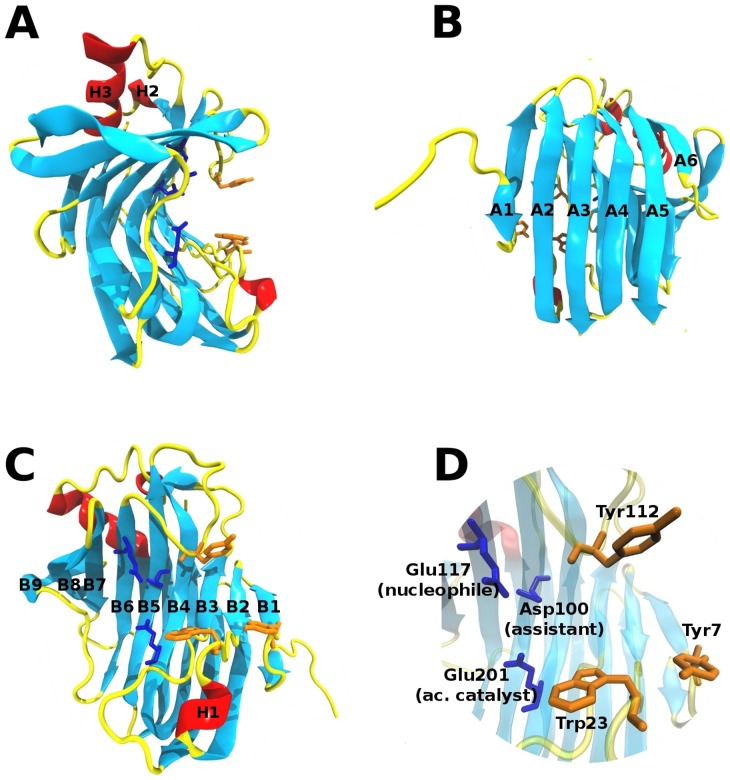
3D structure of the *T. harzianum* endoglucanase 3. (A) The β-jelly roll structure of the endoglucanase 3 from *Trichoderma harzianum*. (B, C) Same as A, after a 90° rotation upon the *y* axis. (D) Closer view of the catalytic cleft, showing the catalytic triad in blue and the three aromatic residues that are located at the cleft entrance, in orange.

The three-dimensional structure of the ThEG3 was determined by X-ray crystallography and is shown in the Figure 1A. The structure is composed by two leaflets of anti-parallel β-sheets, in which the convex and concave parts are formed by six (A1–A6) and nine (B1–B9) strands, respectively, as depicted in Figures 1B and 1C. The β-strands are connected via several loops and three α-helices (H1, H2 and H3; Figure 1A, 1C). The concave part of the leaflets constitutes the catalytic cleft, which binds to the cellulose chains during the hydrolysis. Figure 1D shows the catalytic residues Asp100, Glu117, and Glu201, strictly conserved in the glycosyl hydrolase family 12 [12], [45]. The distance of 5.6 Å between Glu117 and Glu201 is typical for the nucleophile/acid catalyst pairs involved in the hydrolysis mechanism that gives rise to retention of the anomeric configuration of the reaction product [30]. Figure 1D also highlights the three aromatic residues located in the loops that for the ‘thumb’ and ‘fingers’ of the catalytic cleft of enzyme [46]. Our MD simulations suggest that these residues should play an essential role to the efficiency of the hydrolytic catalysis.

### ThEG3 vs. TrEG3: structure and dynamics

**Figure 2:**
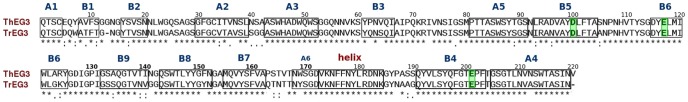
Comparison of the primary structure of the ThEG3 and TrEG3. Alignment of the primary sequence of ThEG3 and TrEG3 with the catalytic triad residues marked in green. The symbols are the adopted by the ClustalW tool (http://www.clustal.org/clustal2/), in which the asterisk indicates fully conservation of the residue, the colon indicates residues with strongly similar properties, and period, residues of weakly similar properties.

**Figure 3:**
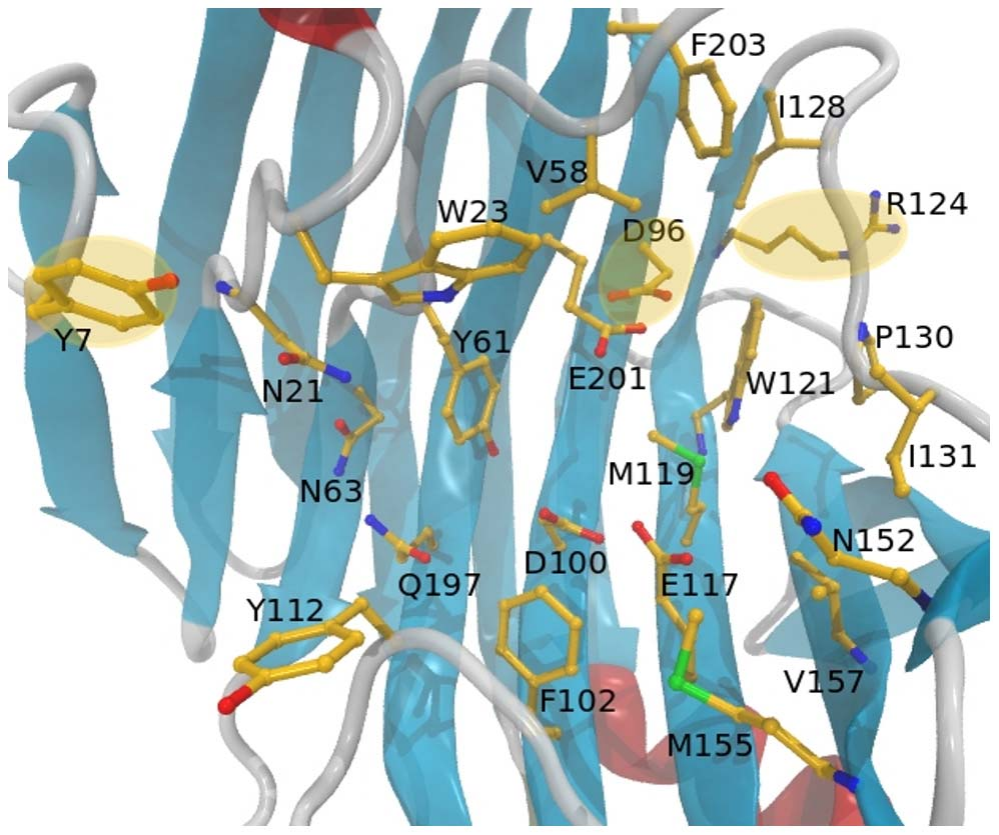
Substrate binding cleft of the ThEG3. Some important residues were drawn explicitly. The highlighted residues differ from the homologue, TrEG3. Residues Tyr7, Asp96, and Arg124 in ThEG3 are replaced by Trp7, Asn95, and Lys123 in TrEG3.

The ThEG3 is composed by 220 residues and has high primary sequence identity (83%) with TrEG3, with just two additional residues (Figure 2). These residues are Val220, localized in the C-terminus of the enzyme, and Gly13, which renders the loop connecting the B1 and B2 strands a bit longer than that of TrEG3. The TrEG3 structure has been described in great detail previously [12] and most of its structural features are similar to that of ThEG3. However, we point out a few differences in the primary structures that may be relevant to the enzymes function. Figure 3 shows residues that are solvent exposed in the substrate binding cleft of ThEG3. Like TrEG3, there is an evident row of hydrophobic residues in one of the edges of the cleft, formed by Tyr7, Trp23, Val58, Phe203, and Ile128. Interestingly, in the GH family 12, a tryptophan residue is most frequently found instead of tyrosine at the amino acid position #7. This is the case of TrEG3, for instance. The effects of the Tyr7/Trp7 substitution are not entirely clear. It is likely that Tyr7 would hold somewhat weaker interactions with the substrate due to the smaller hydrophobic contact area relative to Trp7. This is consistent with the recently reported Michaelis-Menten kinetics which yield *K*_M_∼21.4 g/L for ThEG3 [47], suggesting that the substrate binding affinity to ThEG3 is roughly 14 times smaller than to TrEG3 (*K*_M_∼1.5 g/L) [48].

The deeper surface of the crevice, the ‘palm’, exposes polar amino acids such as Asn21, Asn63, and Gln197 to the solvent. Close to the acid catalyst Glu201, ThEG3 has another acid residue, Asp96, whereas TrEG3 has Asn95. Another difference found between the two homologues is that the residue Arg124 in ThEG3 replaces Lys123 in TrEG3. This substitution leads to important differences in the flexibility of the two enzymes in the vicinity of the B9 strand, according to the MD simulations, as described below.

**Figure 4:**
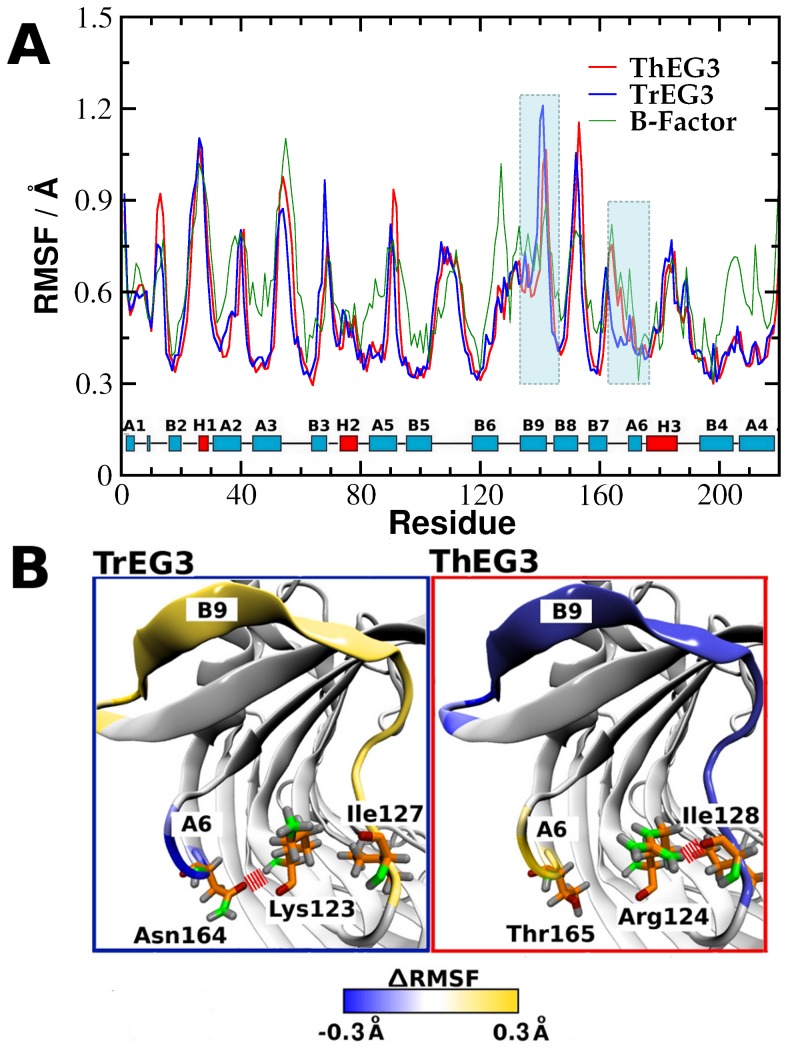
Flexibility of ThEG3 and TrEG3. (A) The mobility profile along the primary sequence of ThEG3 and TrEG3, expressed via the root mean square fluctuations (RMSF) of the residues relative to their average positions calculated over each 150 ps stretch of the simulations. For comparison, the RMSF of the ThEG3 residues calculated from the crystallographic B-factors is also shown (green line). (B) Regions that display relative higher (yellow) and lower (blue) mobility in the TrEG3 and ThEG3. Also shown are the residues involved in interactions responsible for the difference in mobility between the two homologues.



We analyzed the flexibility profile of both ThEG3 and TrEG3 in the MD simulations. The root mean square fluctuations (RMSF) of the α-carbons coordinates relative to the average structures obtained from 300 ps simulation blocks along the three independent runs for each system are shown in Figure 4A. The calculated mobility profile is in a good agreement with the experimental values, obtained from the conversion of the crystallographic temperature B-factor of the α-carbons to the root mean square fluctuations [49], [50] according to:

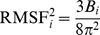
.

It should be noticed that the few differences between the B-factor and the RMSF are not unexpected due to the different conditions in which the data were obtained. MD simulations were performed at room conditions, close to the optimal enzymatic conditions, whereas the crystallographic structure was obtained in much lower temperature and in the crystalline state. The high structural similarity between the two enzymes is reflected in their dynamics, which are also very similar. A few regions display significant differences in mobility, most notably the loops between β-sheets. For instance, the residues in the B9 strand of ThEG3 exhibit lower mobility, whereas residues in the loop that connects strands B7 and A6 present higher mobility relative to TrEG3. These local effects are attributed to the subtle differences in the primary structure of the two enzymes, which alter the interaction between specific residues, as depicted in Figure 4B. In TrEG3, Lys123 interacts with Asn164 via a hydrogen bond, restricting the mobility of the loop between B7 and A6. In ThEG3, Arg124 and Thr165 occupy these positions and are found to interact only weakly with each other. Instead, Arg124 interacts with Ile128, causing stabilization of the B9 strand. This region is the reducing end of the active site, where the reaction product may bind to, contributing to the well-known inhibition of enzymatic activity of cellulases by the reaction product.

### Binding to oligosaccharides

The available crystallographic structures of family 12 glycosyl hydrolases complexed to an inhibitor suggest that the sugar polymers must bind with the non-reducing end up the B1 strand [12]. The recent crystallographic structure of Cel12A from *Thermotoga maritima* [21] bound to a cellotetraose molecule in the active site supports this hypothesis. The four β-glucose residues in this structure occupy the −2, −1, +1, and +2 subsites of the central cleft [51].

**Figure 5:**
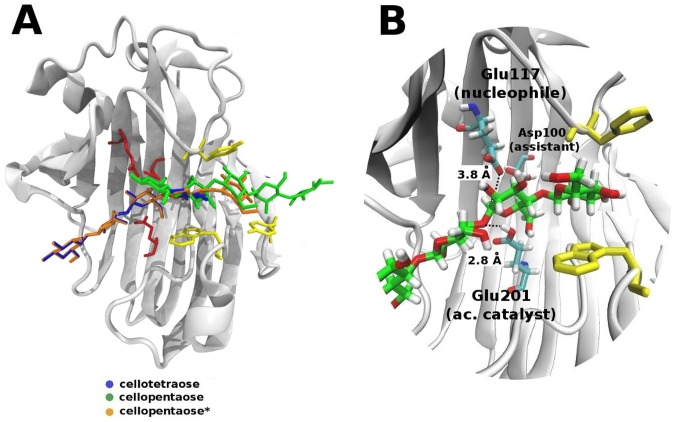
Substrate binding to ThEG3. (A) The initial positions of the substrates in ThEG3 and (B) the interactions of the catalytic residues with cellotetraose (B).

We have built models of ThEG3 bound to cellotetraose and cellopentaose* (ThEG3-tt and ThEG3-pt*) in which the substrates were initially positioned along the −2 to +2 subsites, as shown in Figure 5. This position enables favorable contacts of the sugars with the catalytic triad, which could result in the hydrolysis of the substrates yielding two cellobiose molecules or a cellobiose and cellotriose as reaction products. This docking favors the experimental observation that the cellobiose, not glucose, is the main product of hydrolysis for TrEG3 [11].

Another model structure for the ThEG3-cellopentaose complex is obtained from the crystal structure of the CfCBM-cellopentaose complex, which favors the contact between the sugar and the three aromatic residues at the cleft entrance, at the expense of a better contact with the catalytic residues. The β-glucose residues in this model occupy only the negative subsites −1 to −3. In this case, the distance between the carbonyl oxygen of the nucleophile (Glu117) and the anomeric carbon in the reducing end of the oligosaccharide is as high as 7.6 Å.

**Figure 6:**
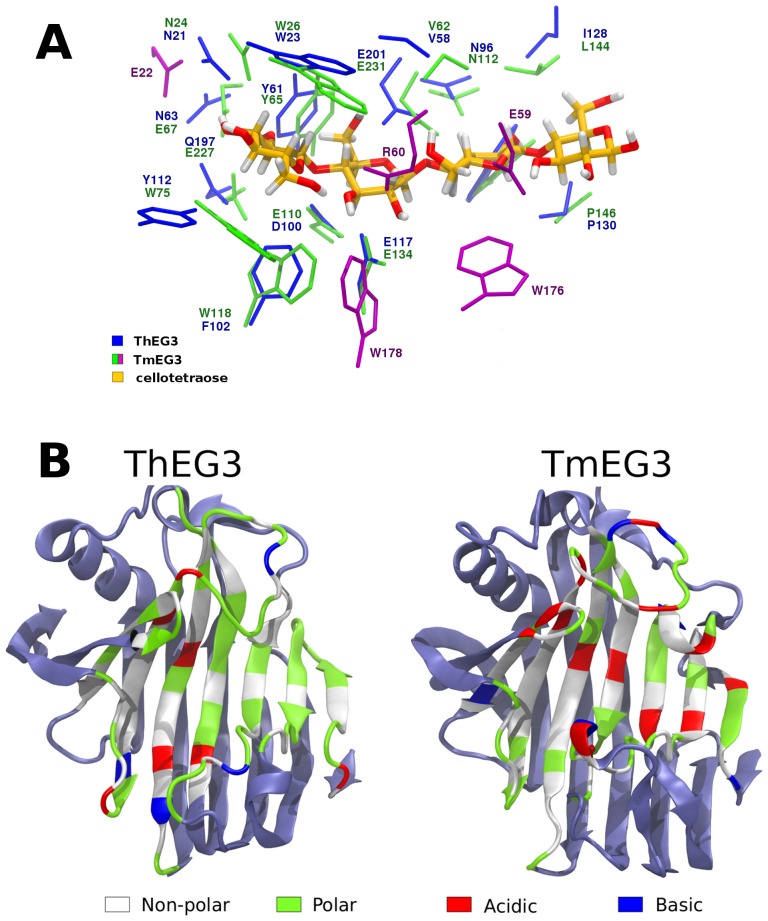
Comparison of the catalytic cleft of ThEG3 and TmEG3. (A) Details of the substrate clefts of ThEG3 and TmEG3 and their interactions with the substrate after structure alignment of ThEG3 with the TmEG3-cellotetraose complex. ThEG3 residues are shown in blue whereas TmEG3 residues are shown in green or purple. The later are residues that have no counterparts in ThEG3. (B) Distribution of residues by chemical nature in the catalytic cleft of both enzymes.

As mentioned in Sec. 2A, the ligand coordinates necessary to build the ThEG3-tt and ThEG3-pt* models were generated from the alignment of the backbone atoms of ThEG3 and TmEG3-tt crystallographic structures. A comparison of the substrate clefts of ThEG3 and TmEG3 is shown in Figure 6, indicating that both enzymes share many common residues important for substrate binding. The Asn63 and Gln197 polar residues in the ThEG3 crevice are substituted by the acidic Glu67 and Glu227 residues in TmEG3. The substitution of polar residues by charged residues is recurrent in thermostable and hyperthermostable enzymes, such as TmEG3 [21]. TmEG3 is also richer in aromatic rings in contact to the substrate. For instance, there are five tryptophan residues near the cellotetraose in TmEG3 (Trp26, Trp75, Trp118, Trp176, Trp178), whereas, in ThEG3, there is only one (Trp23). Residues Glu22, Glu59, Arg60, Trp176, and Trp178 in TmEG3 bear no counterparts in ThEG3 (Figure 6A, in purple). The nature of the residues in the catalytic cleft of both enzymes is compared in Figure 6B.

We use the modeled complexes to gain insights into the behavior of the substrates in the catalytic cleft of the enzyme. During the course of the simulations, the substrates in the modeled ThEG3-tt and ThEG3-pt* structures tend to maintain their initial position relative to the catalytic residues (see Supporting Information). In one of the ThEG3-tt simulations, cellotetraose was unable to keep its initial position and the substrate target sites moved away from the catalytic residues Glu201 and Glu117 after 12 ns along the trajectory. A similar event was observed for cellopentaose in one of the simulations with the ThEG3-pt* structure. However, in this case the substrate was able to re-dock into position and displace again along the trajectory.

As a means of comparison, we monitored ligand-protein distances along simulations of the crystallographic structures of the TmEG3-tt and CfCBM-pt complexes (see Supporting Information). The substrate oscillates much less around its initial position in the cleft during the simulations of the crystallographic TmEG3-tt structure. This is consistent with the fact that the TmEG3 catalytic cleft is richer in charged and aromatic residues, thus being able to establish more persistent contacts with the substrate. In contrast, simulations of the CfCBM-pt crystallographic complex show ligand unbinding from the cleft, similarly to the events observed in some of the ThEG3-tt and ThEG3-pt* runs mentioned above.

**Figure 7:**
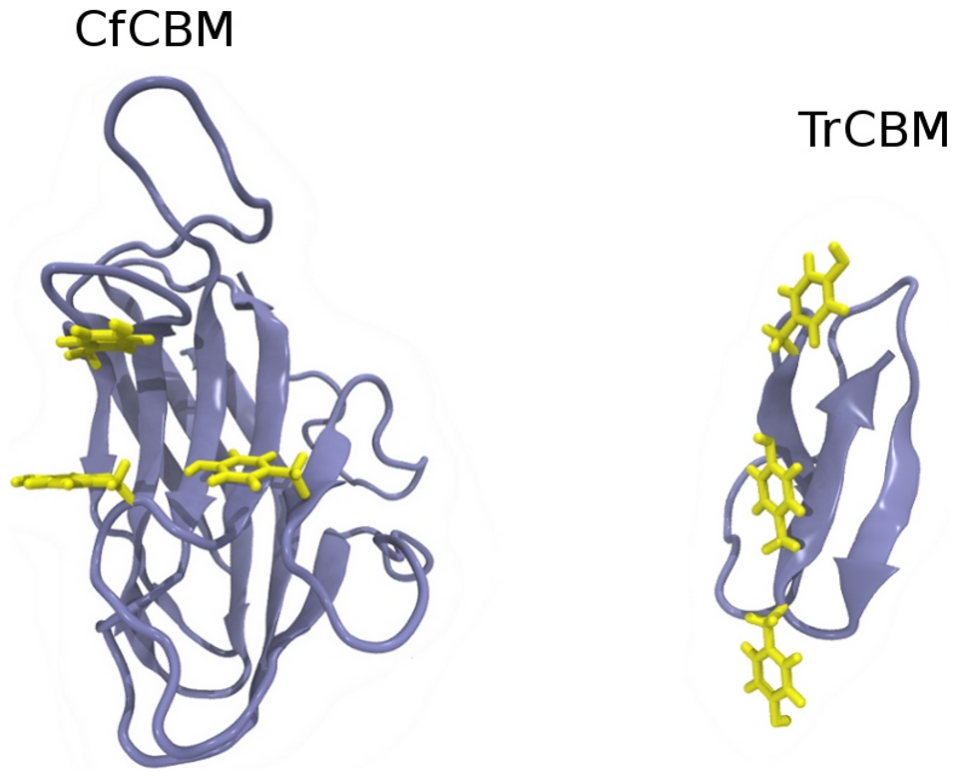
Family 1 and family 4 cellulose binding domains. Structure of cellulose binding domains of the endoglucanase C from *Celulomonas fimi* (left), a family 4 CBM, and of the cellobiohydrolase I from *T. reesei* (right), a family 1 CBM. The hydrophobic residues (yellow) are arranged in different forms, depending on the type of substrate the module preferentially binds to.

The MD simulations also provided valuable information on the interaction of the substrates with the three aromatic residues at the entrance of the catalytic cleft (Tyr7, Trp23, and Tyr112). Aromatic residues, essentially tryptophan and tyrosine, are often found on the loops of catalytic clefts of glycosidases. They can stack with the ring faces of the sugar units, forming carbohydrate-π interactions, and play a pivotal role in protein-carbohydrate recognition mechanisms that are essential in many important biological processes [24], [52], [53]. The importance of aromatic residues in cellulose binding domains of cellulases to the selectivity of the main substrate is widely recognized. A common feature of families 4, 6, 9, and 22 CBMs is the configuration of aromatic amino acids that can ‘sandwich’ the pyranose rings of soluble oligosaccharides or single polysaccharide chains on cellulose amorphous regions [24]. CBMs from members of families 1, 2a, 3, 5, and 10, in turn, present a planar architecture of aromatic residues, which makes binding of these CBMs to crystalline cellulose highly efficient. Figure 7 shows the CBMs of the open cleft family 4 from *C. fimi* (CfCBM) and family 1 cellobiohydrolase I from *T. reesei* as examples of the two distinct arrangements of aromatic residues side chains.

**Figure 8:**
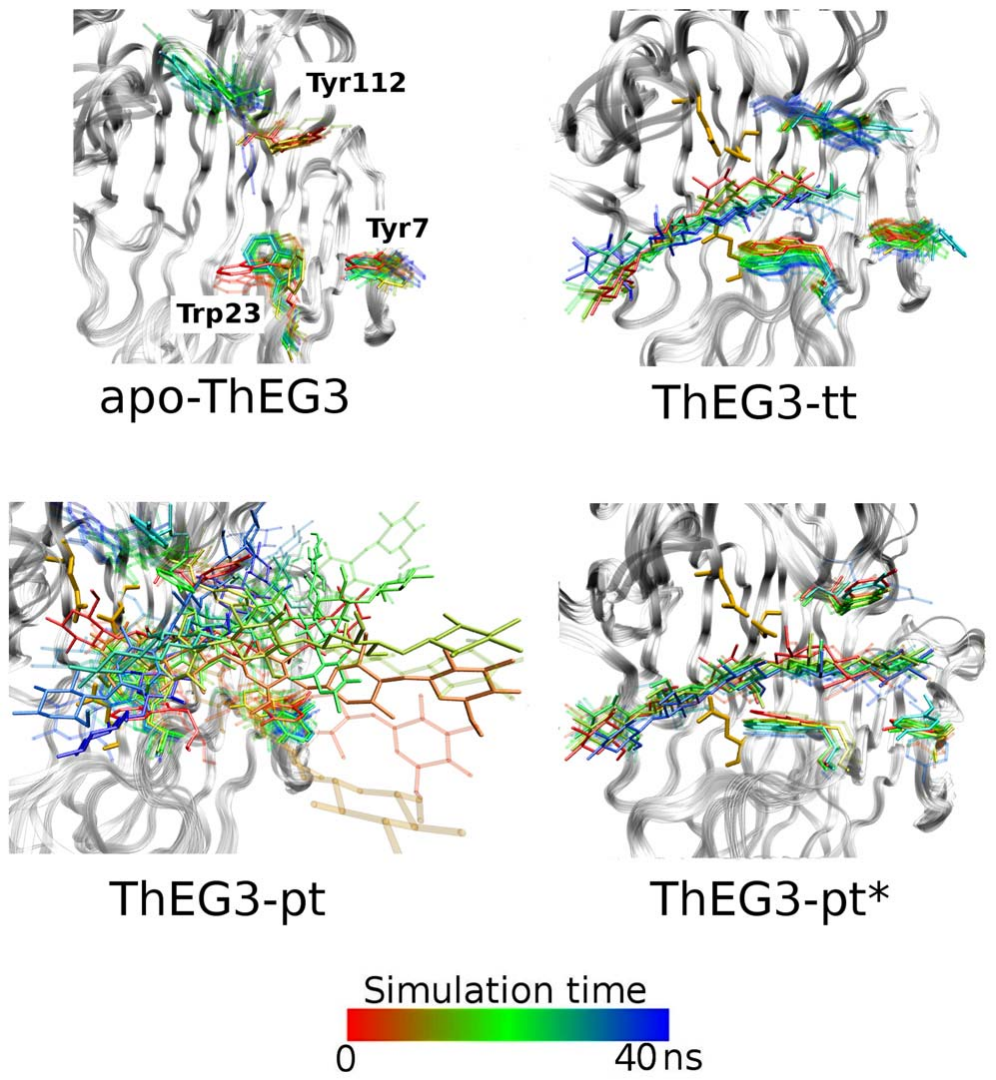
Dynamic picture of the oligosaccharides and aromatic residues nearby. Snapshots from the simulations were superimposed to show the variation on the positions of the aromatic residues Tyr7, Trp23, and Tyr112, and the oligosaccharides: cellotetraose, cellopentaose and cellopentaose*. The aromatic residues and sugar molecules were colored in a scale that varies from red to blue according to the time along the trajectory.

The main position of the substrates relative to the aromatic rings and the catalytic residues are presented in Figure 8, which depicts superposed frames extracted from different stages of the trajectories. In the absence of the ligand, that is, in the apo-ThEG3, the three aromatic amino acids are free to perform larger amplitude motions. The frequent exposure of the ring surface to water may contribute favorably for promoting the first interactions between the protein and the sugar chain that would drive the substrate into the enzyme's catalytic cleft.

In the ThEG3-tt complex, the cellotetraose molecule is adequately positioned with respect to the catalytic triad, but it is not sufficiently long to simultaneously interact with the three aromatic residues at the cleft entrance. In the ThEG3-pt model complex, the substrate position fluctuates considerably and portions of the ligand chain frequently leave the binding cleft. Although, the glucose units of the cellopentaose chain interact with the three aromatic residues at the entrance of the cleft, no glycosidic linkage can reach the catalytic triad. In the ThEG3-pt* complex, however, the initial position and the length of the substrate form a favorable combination that enables the interaction of cellopentaose with both the aromatic and the active-site residues. These features correlate well with the experimentally observed inactivity or low catalytic efficiency of TrEG3 towards the hydrolysis of cellotriose and cellotetraose, as opposed to longer oligosaccharides such as cellopentaose [11]. Therefore, the simulations suggest that the catalytic efficiency of ThEG3 depends on the substrate possessing a minimum length in order to position adequately in the catalytic cleft. This should also apply to other enzymes sharing the same fold and catalytic mechanism.

**Figure 9:**
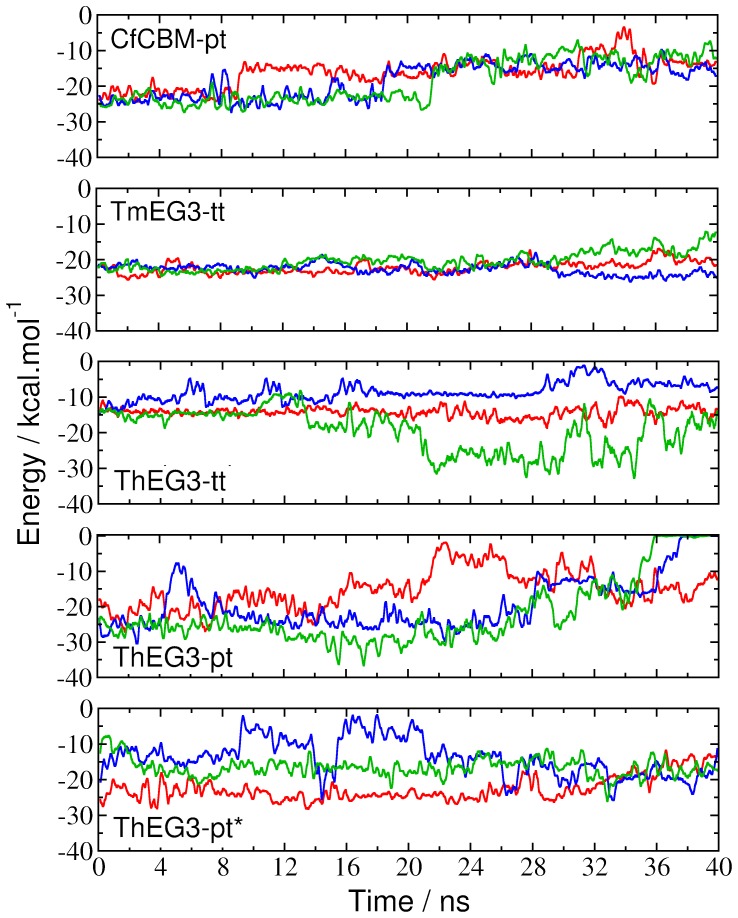
Protein-substrate interaction energies. The time evolution of the interaction energy between the aromatic amino acids Tyr7, Trp23 and Tyr112 and the substrates obtained from simulation of the ThEG3-oligosaccharide models. Also shown are the interaction energies between CfCBM (residues Tyr19, Tyr43 and Tyr85) and TmEG3 (residues Trp26, Trp75, and Trp176) with their corresponding ligands. The curves of different colors correspond to the three independent simulations of each system.

Although one observes events of almost complete detachment of the substrate from the crevice in all simulated models and CfCBM-pt crystal structure, the importance of the aromatic rings in maintaining the substrate-enzyme binding is highlighted by the fact that there remains interactions with one or two of the glucose rings. As shown in Figure 9, the interaction energy between substrate and the three aromatic residues, Tyr7, Trp23, and Tyr112, stays mostly below −10 kcal/mol during the course of the simulations. Except for the TmEG3-tt structure, in which the ligand remains bound to the enzyme for reasons already discussed, all other simulated systems exhibit several instances where the magnitude of the interaction energy drops below 5 kcal/mol.

**Figure 10:**
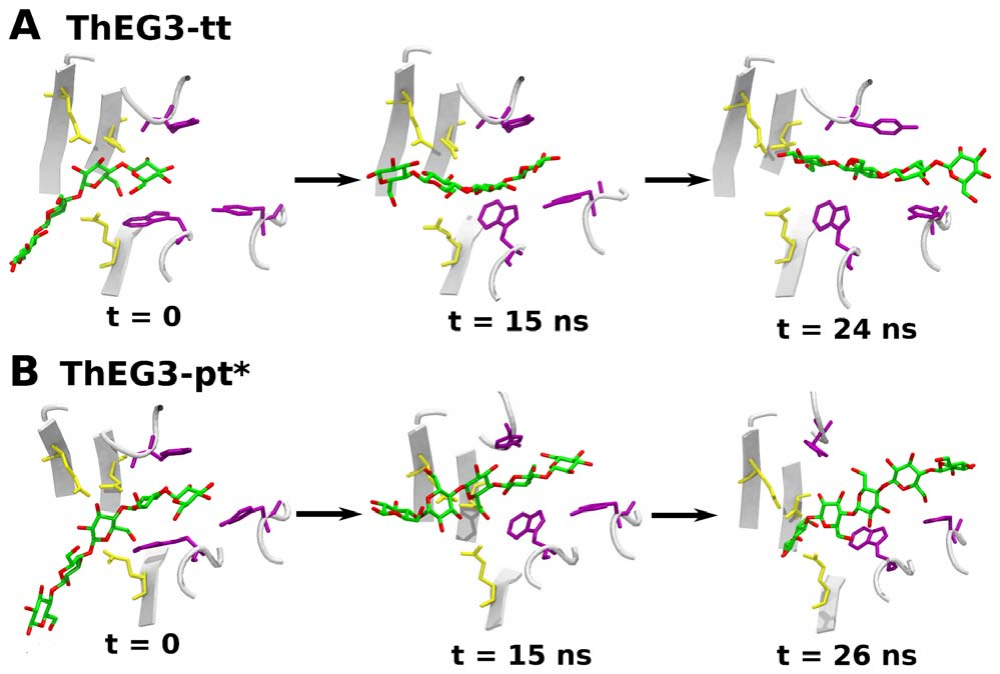
Substrate sliding along the catalytic cleft. Snapshots taken at three different stages along one of the ThEG3-tt (A) and ThEG3-pt* (B) simulations. These events are representative of others that occurred along the simulations for all three modeled systems, in which the substrate remained temporarily bound to the enzyme by just one of the aromatic residues and then fell back in to the cleft.

Close inspection of the trajectories for the ThEG3-tt and ThEG3-pt* models reveals that sometime around 10 ns, the substrate swings away from the binding cleft and from residues Tyr7 and Trp23, but remains connected to the enzyme by the hydrophobic contact with Tyr112, as pictured in Figure 10. Around 15 ns or so, the sugar and the aromatic residues are again strongly interacting. However, at this time, the substrate is not oriented along the crevice. Only at later stages, after 20–30 ns, the substrate fits back into the crevice in a conformation that resembles that of ThEG3-pt. It is worth noting, that a similar substrate docking is found in the crystal structure of the homologue CelB2 from *Streptomyces lividans* (PDB id: 2NLR), in which a cellotriose analogue is bound in the same region of the enzyme, namely, the −1 and −2 binding sites [54].

The events just described suggest that the oligosaccharide chains can slide along the catalytic cleft without completely leaving the crevice due to the arrangement of the aromatic residues in the ThEG3 cleft entrance. That is, when the substrate returns positioned parallel to the cleft in the final stages of the simulation, it turned out displaced by two binding sites relative to its initial position. The processive release of cellobiose from cellulosic substrates is frequently found in exoglucanases [55], [56]. This mechanism is often related to the tunnel-shaped conformation of the catalytic cleft, where the substrate is progressively cleaved from the endpoint of the chain. In contrast, endoglucanases exhibit open catalytic clefts and randomly cleave cellulose chains at arbitrary positions [57]. Exceptionally, few cellulases were identified as processive endoglucanases [58], [59] and their processivity is found to be independent of their cellulose binding modules. The present simulations may provide an interesting starting point to explore the molecular basis of the processivity of endoglucanases, which is yet poorly understood. In this regard, our simulations and analysis are very preliminary and a much more thorough investigation would be necessary to elucidate such mechanisms.

### CBM motifs in the catalytic core

**Figure 11:**
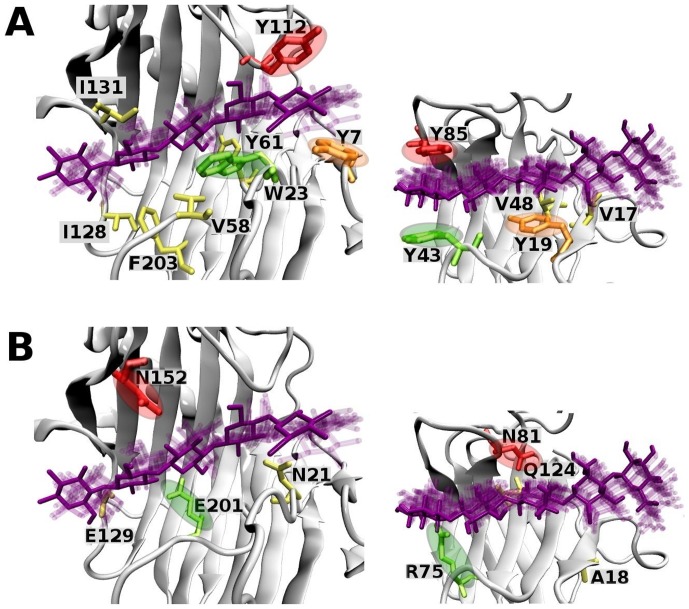
Hydrogen bonds and hydrophobic contacts. (A) The residues of ThEG3 (left) and CfCBM (right) in hydrophobic contact with a cellopentaose molecule and (B) residues that hydrogen bond to the protein at least 10% of the simulation time. Circles of same color highlight residues that are correspondent in position and function in the protein-substrate interactions in the enzyme (left) and CBM (right) structures.

Finally, comparative analysis of the structure and MD simulations of ThEG3 and CfCBM complexed with cellopentaose reveal that several residues play similar roles in their interaction with the substrate, which strongly suggest that the ThEG3 bears CBM function in its catalytic domain. Figure 11 shows the residues that maintain persistent hydrophobic contacts (Fig.11A) with the substrate and also residues that engage in hydrogen bonding with the cellopentaose molecule for at least 10% of the simulation time (Fig.11B) in the ThEG3-pt* complex (see Supporting Information for further details). The three aromatic residues identified as important hydrophobic anchors for the substrate binding in ThEG3 (Tyr7, Trp23 and Tyr112), mimic residues Tyr19, Tyr43, and Tyr85 in terms of position and function in the CfCBM structure. Residues Asn152 and Glu201, which hydrogen bond with the sugar molecule in the ThEG3-pt* simulations, are reciprocated by Asn81 and Arg75 in CfCBM. In total, there are considerably more hydrophobic contacts in ThEG3 than in CfCBM. This is not surprising, since ThEG3 combines, within a single structure, features of a catalytic domain and a CBM-like substructure. These results suggest that the absence of a cellulose-binding module in endoglucanases 3 is partially compensated by the presence of a CBM-like cluster of residues. In addition, these findings immediately give new guidelines for enzyme engineering. It might be possible to introduce the CBM function in the catalytic core, even for those enzymes that naturally contain the CBM. Thereby, the enzyme affinity by the substrate, the concentration of the enzyme on the cellulose surface and even disruption function could be improved or modulated by introducing CBM motifs in the catalytic domain.

## Concluding Remarks

We solved the crystallographic structure of endoglucanase 3 from *Trichoderma harzianum* at 2.07 Å resolution and performed molecular dynamics simulations on this new structure with the aim to shed more light on the Cel12A dynamics and its binding mechanism oligosaccharides. Model structures of this enzyme bound cellotetraose and cellopentaose substrates were generated using available crystallographic structures of proteins of similar folds bound to these substrates as templates. The simulations suggest that a CBM-like cluster of key residues located in the loops at one end of the catalytic cleft is responsible for recognizing and binding the polymeric substrate. This region is spatially distant from the catalytic residues in the active site. The success of productive substrate binding and catalytic efficiency, therefore, requires oligosaccharide chains of a minimum length, such that the residues of the catalytic triad and the CBM-like cluster of the aromatic residues may be simultaneously reached. These results provide a molecular basis for the experimental observation that Cel12A does not efficiently hydrolyzes short oligosaccharides such as cellotriose in addition to suggest strategies to engineer proteins aiming to improve interactions of GHF12 enzymes with cellulosic substrates.

## Supporting Information

Figure S1 **Root mean square deviations of the backbone atoms from the the crystallographic structures.** Root mean square displacements of the backbone atoms for the ThEG3-substrate models as well as the CfCBM and TmEG3 liganded crystallographic structures along the simulations. The crystal structures were used as reference. The lines of different colors correspond to the independent simulations for each system. In order to show the stabilization of the simulations, the first eight residues that form the N-terminal loop of the ThEG3were not considered, since this portion presents too high mobility. For the same reason, the first 14 residues of CfCBM were not considered in this analysis. RMSD values of CfCBM are significantly higher than the other proteins because this CBM possesses many large and mobile loops, one of them is 11 residues long. Abbreviations: tt-cellotetraose, pt–cellopentaose, pt*-cellopentaose*.
(TIFF)

Figure S2 **Time evolution of the distances between catalyst residues and substrate atoms.** The time evolution of the distances between the acid catalyst (Glu201 in ThEG3 and Glu134 in TmEG3), and a glycosidic oxygen (dotted lines), and between the nucleophile (Glu117 in ThEG3 and Glu231 in TmEG3), and a C1 atom in the glucose unit (full lines). We have selected the O and C1 atoms in the closest glycosidic bonds (the second glycosidic bond in cellotetrose and cellopentaose* and the first one in cellopentaose, counting from the reducing end of the sugar chain). And we have considered the acidic hydrogen from the acid catalysts and the carboxylic oxygen atoms from the nucleophiles. For CfCBM, we have computed the distances between the residues Gln124 (amide oxygen in the lateral chain) and a hydroxyl hydrogen (from C6 of the second glucose unit) and between Gln128 (amide hydrogen in the lateral chain) and a hydroxyl oxygen (from C3 of the first glucose unit). The curves of different colors correspond to the three independent simulations of each system.
(TIFF)

Table S1 Frequencies of hydrophobic contacts and hydrogen bonds in the simulations of EG3Th-pt* and CBMCf-pt.
(PDF)
