# Supplementary figures and images for: X-ray Structure and Molecular Dynamics Simulations of Endoglucanase 3 from Trichoderma harzianum: Structural Organization and Substrate Recognition by Endoglucanases That Lack Cellulose Binding Module
Source: PLoS One. 2013 Mar 14;8(3):e59069. doi: 10.1371/journal.pone.0059069 (PMC3597598; doi:10.1371/journal.pone.0059069)

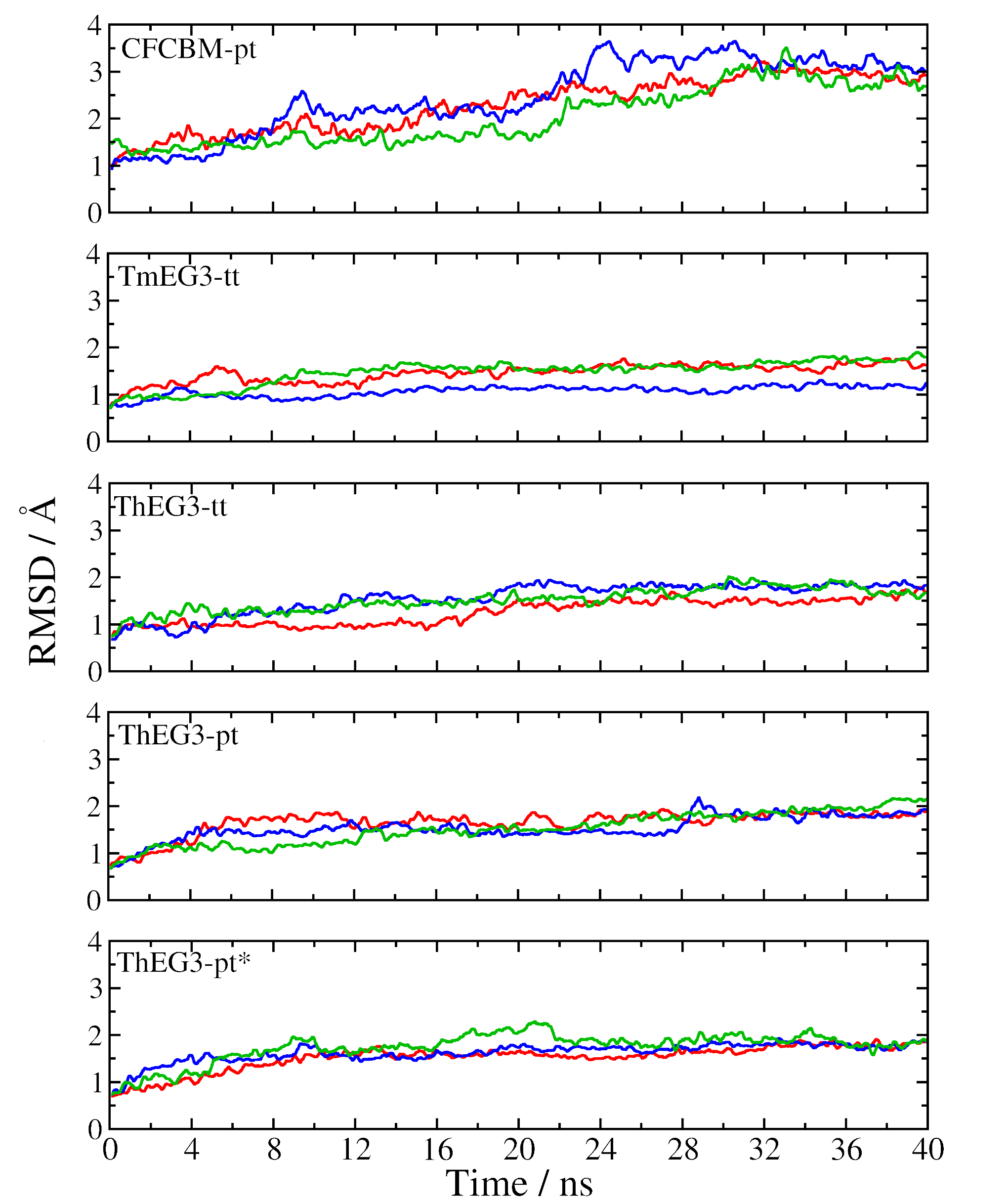

Supplement: Figure S1 — Root mean square deviations of the backbone atoms from the the crystallographic structures. Root mean square displacements of the backbone atoms for the ThEG3-substrate models as well as the CfCBM and TmEG3 liganded crystallographic structures along the simulations. The crystal structures were used as reference. The lines of different colors correspond to the independent simulations for each system. In order to show the stabilization of the simulations, the first eight residues that form the N-terminal loop of the ThEG3were not considered, since this portion presents too high mobility. For the same reason, the first 14 residues of CfCBM were not considered in this analysis. RMSD values of CfCBM are significantly higher than the other proteins because this CBM possesses many large and mobile loops, one of them is 11 residues long. Abbreviations: tt-cellotetraose, pt–cellopentaose, pt*-cellopentaose*. (TIFF) [file pone.0059069.s001.tif]

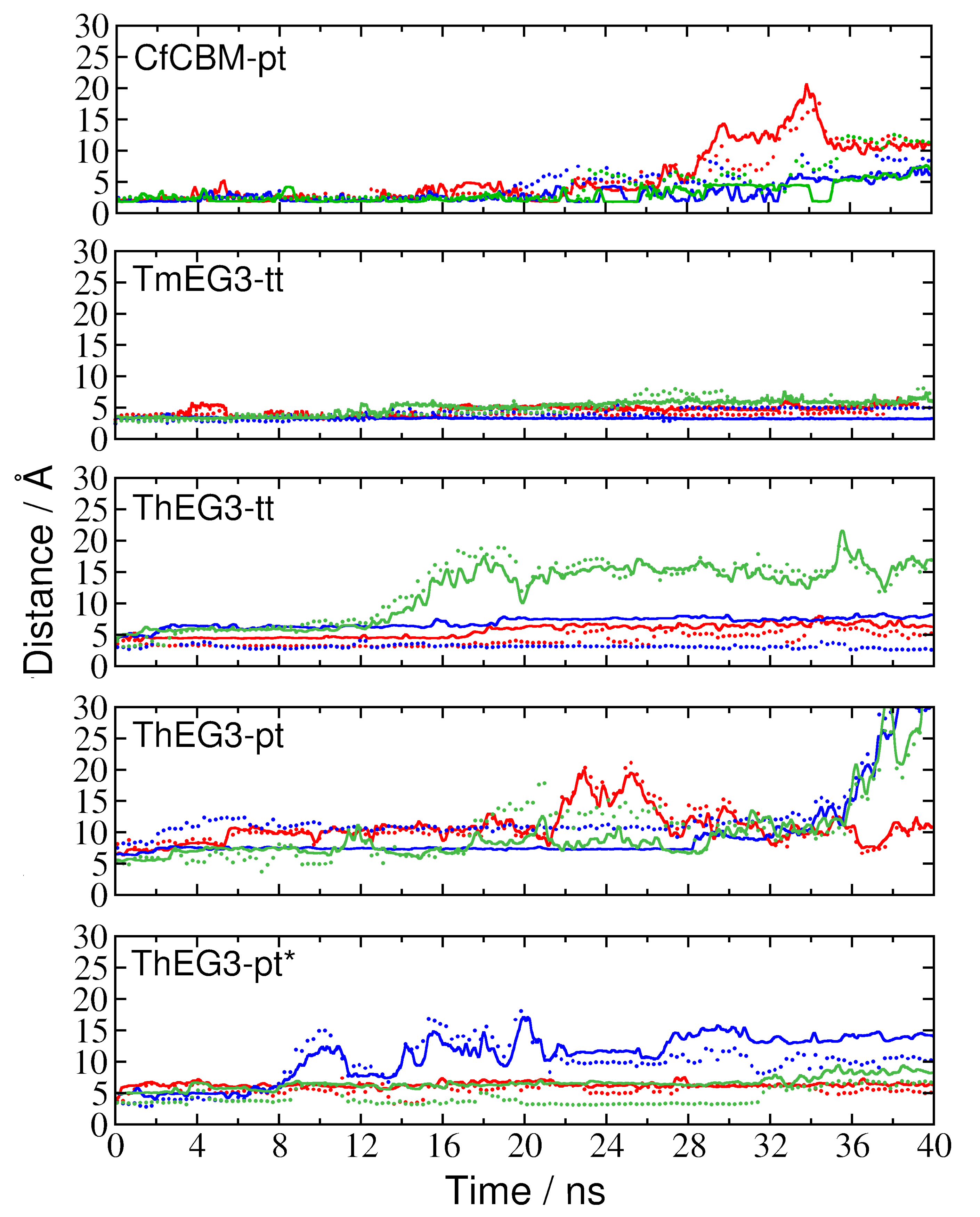

Supplement: Figure S2 — Time evolution of the distances between catalyst residues and substrate atoms. The time evolution of the distances between the acid catalyst (Glu201 in ThEG3 and Glu134 in TmEG3), and a glycosidic oxygen (dotted lines), and between the nucleophile (Glu117 in ThEG3 and Glu231 in TmEG3), and a C1 atom in the glucose unit (full lines). We have selected the O and C1 atoms in the closest glycosidic bonds (the second glycosidic bond in cellotetrose and cellopentaose* and the first one in cellopentaose, counting from the reducing end of the sugar chain). And we have considered the acidic hydrogen from the acid catalysts and the carboxylic oxygen atoms from the nucleophiles. For CfCBM, we have computed the distances between the residues Gln124 (amide oxygen in the lateral chain) and a hydroxyl hydrogen (from C6 of the second glucose unit) and between Gln128 (amide hydrogen in the lateral chain) and a hydroxyl oxygen (from C3 of the first glucose unit). The curves of different colors correspond to the three independent simulations of each system. (TIFF) [file pone.0059069.s002.tif]
