# Supplementary material for: X-ray Structure and Molecular Dynamics Simulations of Endoglucanase 3 from Trichoderma harzianum: Structural Organization and Substrate Recognition by Endoglucanases That Lack Cellulose Binding Module
Source: PLoS One. 2013 Mar 14;8(3):e59069. doi: 10.1371/journal.pone.0059069 (PMC3597598; doi:10.1371/journal.pone.0059069)
Supplement: Table S1 — Frequencies of hydrophobic contacts and hydrogen bonds in the simulations of EG3Th-pt* and CBMCf-pt. (PDF) [file pone.0059069.s003.pdf]

Table S1: Frequencies of hydrophobic contacts and hydrogen bonds in the simulations of ThEG3-pt* and CfCBM-pt. Only frequencies above 8% are shown.

**ThEG3-pt***	**Hydrophobic Contacts / %**				
	Trp23	Ile131	Tyr112	Phe203	Val58
	100	100	99	98	94
	Tyr61	Ile128	Tyr7	Phe102	Trp121
	94	72	67	42	19
	**Hydrogen Bonds / %**				
	Gly129	Glu201	Asn21	Asn152	
	41	36	26	17	
**CfCBM-pt**	**Hydrophobic Contacts / %**				
	Tyr19	Val17	Val48	Tyr43	Tyr85
	100	100	98	100	98
	**Hydrogen Bonds / %**				
	Gln124	Asn81	Ala18	Arg75	
	37	33	34	29	

Hydrophobic contact:

It was considered that the hydrophobic contacts exist if the distances between carbon atoms in the lateral chains of hydrophobic residues (Val, Leu, Met, Ile, Pro, Trp , Phe and Tyr) and atoms in glycosidic rings "backbone" are smaller than 5 Å.

Hydrogen bonding:

The adopted criteria to consider a hydrogen bond were *D-A* distance less than 3 Å, *D-H-A* angle less than 20°, being *D*, the donor and *A*, the acceptor of proton.
